# Layperson-Supported, Web-Delivered Cognitive Behavioral Therapy for Depression in Older Adults: Randomized Controlled Trial

**DOI:** 10.2196/53001

**Published:** 2024-03-04

**Authors:** Xiaoling Xiang, Jay Kayser, Skyla Turner, Samson Ash, Joseph A Himle

**Affiliations:** 1 School of Social Work University of Michigan Ann Arbor, MI United States

**Keywords:** internet-based cognitive behavioral therapy, iCBT, digital mental health interventions, older adults, depression

## Abstract

**Background:**

Depression is the most prevalent mental health condition in older adults. However, not all evidence-based treatments are easily accessible. Web-delivered cognitive behavioral therapy (wCBT) facilitated by laypersons is a viable treatment alternative.

**Objective:**

This randomized controlled trial aims to evaluate the efficacy of a novel wCBT program, Empower@Home, supported by trained lay coaches, against a waitlist attention control. Empower@Home is among the very few existing wCBT programs specifically designed for older adults. The primary objective was to assess the efficacy of the intervention compared with attention control. The secondary objective was to evaluate the program’s impact on secondary psychosocial outcomes and explore potential change mechanisms.

**Methods:**

Older adults (N=70) were recruited via web-based research registries, social media advertisements, and community agency referrals and randomly assigned to either the intervention or control group in a 1:1 allocation ratio. The intervention group received access to Empower@Home, which included 9 web-delivered self-help lessons and weekly telephone coaching sessions by a trained layperson over 10 weeks. The control group received weekly friendly phone calls and depressive symptom monitoring. The primary clinical outcome was the severity of depressive symptoms assessed using the Patient Health Questionnaire-9. The secondary clinical outcomes included anxiety, anger, social isolation, insomnia, pain intensity, and quality of life. Linear mixed modeling was used to determine the treatment effects on depression, and 2-tailed *t* tests were used to assess within-group changes and between-group differences.

**Results:**

Most participants in the intervention group completed all 9 sessions (31/35, 89%). The usability and acceptability ratings were excellent. The intervention group had a large within-group change in depressive symptoms (Cohen *d*=1.22; *P*<.001), whereas the attention control group experienced a medium change (Cohen *d*=0.57; *P*<.001). The between-group effect size was significant, favoring the intervention group over the control group (Cohen *d*=0.72; *P*<.001). In the linear mixed model, the group-by-time interaction was statistically significant (b=–0.68, 95% CI –1.00 to –0.35; *P*<.001). The treatment effects were mediated by improvements in cognitive behavioral therapy skills acquisition; behavioral activation; and satisfaction with the basic psychological needs of autonomy, competence, and relatedness. Furthermore, the intervention group showed significant within-group improvements in secondary psychosocial outcomes, including anxiety (*P*=.001), anger (*P*<.001), social isolation (*P*=.02), insomnia (*P*=.007), and pain (*P*=.03). By contrast, the control group did not experience significant changes in these outcome domains. However, the between-group differences in secondary outcomes were not statistically significant, owing to the small sample size.

**Conclusions:**

Empower@Home, a wCBT program supported by lay coaches, was more efficacious in reducing depressive symptoms than friendly telephone calls and depression symptom monitoring. Future studies should examine the effectiveness of the intervention in community and practice settings using nonclinician staff already present in these real-world settings as coaches.

**Trial Registration:**

ClinicalTrials.gov NCT05593276; https://clinicaltrials.gov/ct2/show/NCT05593276

**International Registered Report Identifier (IRRID):**

RR2-10.2196/44210

## Introduction

### Background

Depression is the most prevalent mental health condition among older adults and is particularly common among those with chronic physical health conditions and functional limitations [1]. Although evidence-based pharmacological and nonpharmacological treatments exist, not every option is readily accessible to all populations of older adults [2,3]. For example, older adults with mobility difficulties face significant logistical barriers to accessing office-based treatments in addition to common access barriers such as cost, provider shortages, and stigma [2].

Web-delivered cognitive behavioral therapy (wCBT), also known as internet-based cognitive behavioral therapy or computerized cognitive behavioral therapy, is a promising option for addressing the unmet mental health needs of older adults. wCBT, initially introduced in the 90s via CD-ROM manuals, now typically presents cognitive behavioral therapy (CBT) principles through a combination of audio, video, and text elements hosted on dedicated websites or apps. The advantages of wCBT include its low cost, efficiency, convenience, and accessibility [4]. Moreover, preprogrammed components in wCBT can minimize variability between trials and dissemination, thus maintaining greater fidelity to treatment protocols compared with the potential variability or *drift* seen in face-to-face CBT sessions [5].

Although wCBT is promising, its real-world impact can be hampered by low user engagement [6]. Common issues with wCBT programs include dense text, academic oriented content, and a tendency to adopt a *one size fits all* approach [7]. Furthermore, there is a scarcity of wCBT programs specifically designed for older adults, with only a handful having undergone development and testing [8-10]. Although some generic wCBT programs have demonstrated effective depression reduction among older adults [11-13], our assessments of underserved older adult populations, encompassing individuals with low income, limited literacy, technological challenges, and disabilities, have revealed many usability and engagement problems [14,15]. For wCBT to truly benefit older adults, there is a pressing need to address these issues with more inclusive, user-friendly, and engaging programs.

To our knowledge, wCBT programs tailored to the needs of older adults and readily available for US consumers are lacking. We addressed this shortage and developed Empower@Home in response to the scarcity of wCBT programs tailored to the needs of older adults in underserved communities. The design process of Empower@Home, detailed elsewhere [16], was grounded in a user-centered and community-engaged approach. The final program included 9 web-delivered sessions grounded in CBT principles, presented via a custom-made website with a user-friendly interface. Along with the web sessions, we provided a large print user workbook featuring session summaries, home practices, and wellness resources. A unique aspect of our approach is the infusion of entertainment through a character-driven storyline. This story centers on Jackie, a homebound older adult character presented as a 74-year-old woman who navigates health challenges typical of the target demographic. Her experiences are depicted in an animated series shaped by stakeholder input. The web program was enhanced with the inclusion of “Empower Coaches,” laypersons trained to support users. Research has shown that wCBT interventions with human support are more efficacious than those without human assistance [17-19]. Layperson coaches offer an alternative to relying on scarce gerontological mental health professionals.

During usability and field tests, Empower@Home showed superior usability compared with 2 evidence-supported and commercially available wCBT programs [16]. Findings from an uncontrolled trial demonstrated a medium effect size for depression reduction in older adults with mild depression and a large effect size in those with moderate depression [14]. However, the intervention is yet to be assessed against a control condition.

### Objectives

This study aims to evaluate the efficacy of layperson-supported Empower@Home for depression in older adults through a randomized controlled trial (RCT). The primary hypothesis was that the treatment group would experience a significant reduction in depressive symptoms compared with the attention control group. In addition, this study aims to investigate the impact of the intervention on secondary clinical outcomes, including anxiety, loneliness, pain severity, and overall disability burden, with the hypothesis that the treatment group would show greater improvements in these areas than the control group. Furthermore, this study intends to explore potential mediators to gain a deeper understanding of the mechanisms responsible for the expected reductions in depressive symptoms.

## Methods

### Ethical Considerations

**Figure 1:**
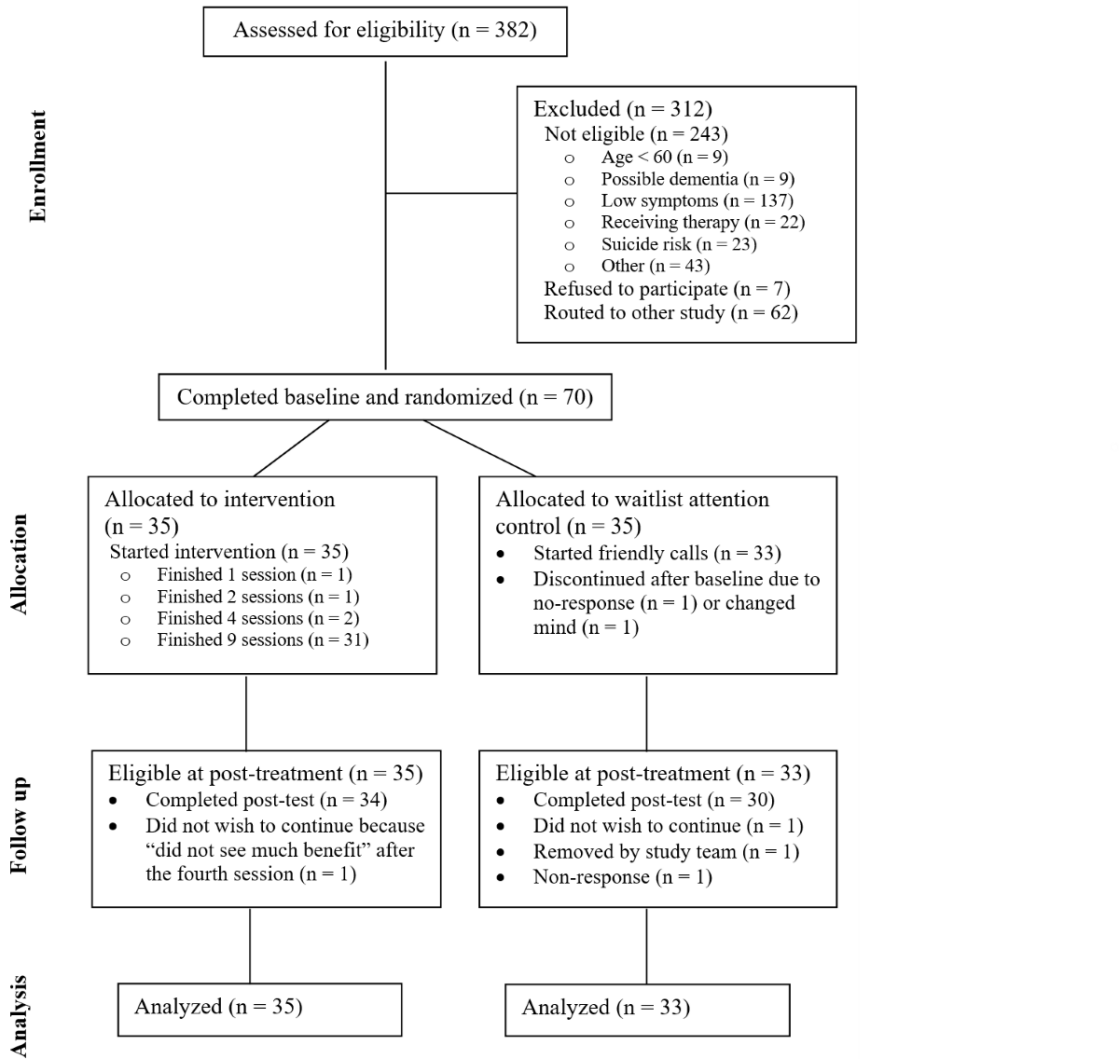
Participant flow through the study.

The study protocol has been published elsewhere [3]. An abbreviated description of the study methods has been provided in this study to inform the readers. The study was approved by the University of Michigan Institutional Review Board–Health Sciences and Behavioral Science (HUM00212950) and was registered on ClinicalTrials.gov (NCT05593276) on October 24, 2022. Participants were eligible to receive up to US $100 for participation in the study. Figure 1 illustrates the flow and allocation of the participants. After screening, eligible participants were invited to provide their informed consent. They completed the consent document by phone and were provided with a mailed copy upon providing verbal consent. Before beginning the first session, the participants provided their consent on the website hosting the program.

### Study Design

The study adopted a parallel RCT design, with participants randomly assigned to either Empower@Home supported by trained lay coaches or a waitlist attention control group, maintaining a 1:1 allocation ratio. The allocation followed a computer-generated random sequence. The principal investigator (XX) generated the sequence, whereas the project coordinator (ST) managed the actual allocation. Other research staff, who were uninformed of the sequence and allocation decision, conducted screening assessments. Although it was not feasible to blind participants to their conditions owing to the intervention’s content and study design, research staff conducting follow-up assessments were blinded.

### Participant Recruitment

Participants were recruited from research volunteer registries, social media advertisements, and referrals from community agencies. To qualify, participants were required to be able to read and speak English, reside in Michigan, be aged at least 60 years, and have elevated depressive symptoms at screening (as indicated by a score of ≥8 on the Patient Health Questionnaire-9 [PHQ-9]). Participants were excluded if they had probable dementia, a psychotic disorder, moderate to high risk of suicide, a terminal illness, a current substance use disorder, or uncorrected severe vision impairment (eg, blindness) or if they were receiving or planning to receive psychotherapy during the trial. Lack of device ownership or internet access was not an exclusion criterion. Participants without a computer or internet access were provided with a cellular tablet at no cost during the trial.

### Procedures

Potential participants identified from volunteer registries and social media (Facebook) advertisements were directed to a web survey to complete a general screening survey used across multiple studies. Facebook advertisements were directed across all areas of Michigan to users aged ≥60 years. The research team contacted individuals who met the preliminary eligibility criteria up to 3 times for further phone screening. To accommodate potential internet access issues, referrals from community agencies skipped the web screener and were called directly by the research team. Recruitment was completed between January and June 2023.

Assessments were conducted over the phone, with a baseline assessment occurring within 2 weeks preceding the start of the 10-week intervention and a posttest assessment within 2 weeks following the intervention or waiting period. Participants were eligible to receive a total compensation of up to US $100 for completing research assessments, comprising US $30 for the baseline assessment, US $50 for the posttest assessment, and US $20 for a 10-week follow-up (not discussed in this paper). We emphasized that this compensation was solely contingent on completing the research assessments and had no connection to participation in the intervention or coaching. This distinction was repeated multiple times by our consent staff and coaches to ensure clarity regarding payment structure.

Participants in the waitlist control group were contacted by trained research staff with educational backgrounds similar to those of the Empower Coaches who supported the participants in the treatment group. These staff members (N=3) conducted weekly friendly phone calls, averaging 17 minutes each, and administered depression assessments using the PHQ-9 every 2 weeks. These “friendly callers” followed a structured call guide with a bank of questions to guide each call, such as “How has your week been?” or “What was the highlight of your week?” Callers were asked to prioritize empathetic listening during the calls. After their posttest assessment, the control participants were offered the Empower@Home program.

We used a waitlist group with attention control to differentiate between the effects of human interaction and the wCBT program itself. This approach was crucial for assessing whether improvements were because of the program or merely the result of coach-participant interactions. Ethically, we offered the intervention to the waitlist control group after the posttest assessment, as many participants needed treatment and were not service connected. A comparison with another wCBT program was not ideal, as our goal was to evaluate the efficacy of our novel program and not to prove its superiority. In addition, the efficacy of other wCBT programs in the target population was uncertain. In summary, this design enabled a clear assessment of our intervention’s efficacy in improving mental health outcomes in older adults.

All 35 participants in the treatment group started the wCBT program and were included in the analysis. In the control group, 2 participants withdrew from the study after the baseline assessment and before the initiation of friendly calls, and they were excluded from the analysis. This resulted in 33 participants in the control group included in the analysis. Notably, 1 participant from the control group was removed from the study because of a recent significant loss and pronounced grief concerns, as assessed by our clinical social worker. It was determined that this participant required more immediate assistance than the program was designed to provide, and a removal decision was made according to our protocol.

### Intervention

Empower@Home contains 9 sequenced sessions, drawing from the CBT manual for working with older people from the study by Gallagher-Thompson and Thompson [20] and the Behavioral Activation Manual from the study by Lejuez et al [21], with adaptations to address the needs of older adults. Specific adaptations, development, and details of the program content, including the coaching component, have been documented elsewhere [14,16]. Essentially, users accessed self-help therapeutic tools and lessons through a dedicated website using a pregenerated username and password found in their user workbook for easy reference. Once logged onto the website, they navigated the web-delivered sessions through a blend of brief videos, narrated text pages, short exercises, and offline home practices. Each session was designed to be completed independently in approximately 20 to 30 minutes. Users completed sessions in their homes and had the option to complete sessions during their weekly coaching calls, based on their preferences. The participants were encouraged to complete 1 session weekly. Apart from the coaching calls, participants did not receive additional prompts to use the web program.

### Coaching

Each participant received support from a trained coach for up to 10 weeks. Coaches adhered to a structured coaching guide developed and refined based on insights from our previous studies [14,16]. This guide is designed to be adaptable, ensuring an optimal balance between the benefits realized and the sources used. For instance, coaches have the flexibility to work through web-delivered sessions with participants who may be less motivated or may frequently face technical issues. By contrast, for those who are highly motivated and rarely encounter technical difficulties, coaches can encourage the independent completion of web sessions and then discuss the content during weekly coaching calls. This tailored approach, grounded in the principle of self-determination [22], seeks to cater to participants’ individual needs while maximizing staff efficiency. Coaches contacted users via SMS text messaging or phone calls to schedule weekly sessions, and reminders were sent to users before the coaching sessions, based on coach discretion. All the coaching sessions were conducted via telephone calls.

The coaches participating in this RCT included undergraduate students in psychology (n=1), students pursuing a master’s degree in social work (n=5), and a master’s-level social worker without licensure or prior psychotherapy experience. Hence, they were all considered lay coaches without specialized mental health expertise. The coaches received group supervision and individualized support from the study’s management team, with a licensed clinical social worker (JK) serving as a clinical backup.

### Measures

#### Primary Clinical Outcome

Depressive symptoms were assessed using the PHQ-9, which is the most commonly used outcome measure in wCBT studies [23] and has been validated for use in remote computerized delivery [24]. Scores of 5, 10, 15, and 20 on the PHQ-9 represent thresholds for mild, moderate, moderately severe, and severe depressive symptoms, respectively. A change of 5 points is deemed clinically significant; a score <10 indicates a partial response, and a score <5 denotes remission [25]. Participants completed the PHQ-9 on up to 7 occasions: at baseline, during 5 biweekly assessments throughout the trial, and at posttest assessment. For the intervention group, the PHQ-9 was completed on the web portal during sessions 1, 3, 5, 7, and 9. The control group was assessed via phone calls during weeks 1, 3, 5, 7, and 9 of the waiting period.

#### Usability, Acceptability, and Engagement Outcomes

Across all measures and participants, data were collected between January and August 2023. Usability was assessed during posttest assessments using the System Usability Scale (SUS), a 10-item scale commonly used to evaluate the usability of websites, software, and other human-machine systems [26]. SUS scores can range from 0 to 100, with scores ≥68 considered above average. The SUS had good internal consistency in the study sample (Cronbach α=.81).

For acceptability assessment following the intervention, we used the Treatment Evaluation Inventory (TEI), specifically designed to evaluate acceptability of depression treatments for older adults [27]. To alleviate respondents’ burden during phone-based assessments, we adapted the original TEI’s 7-point Likert scale responses to a 5-point scale. The modified TEI yields total scores ranging from 11 to 55, with a score ≥32 suggesting positive attitudes toward the treatment. Among the Empower@Home participants, TEI scores exhibited excellent reliability (Cronbach α=.89).

Program engagement was measured by the number of sessions completed, which were logged into the web portal and confirmed by the coaches. These measures were only administered to the intervention group participants, as they were not applicable to the control group.

#### Coaching

The coaches were required to document each coaching call using a structured electronic form. This form captured details such as the call duration, the type of support provided, and a narrative summary of the coaching session. Information collected in the coaching forms was used to calculate the average number and duration of coaching calls, to calculate the percentage of sessions completed independently by participants, and to describe the types of support provided during the coaching calls.

#### Secondary Clinical Outcomes

Secondary outcomes, including anxiety, anger, and social isolation, were measured in baseline and posttest assessments using validated instruments. Generalized Anxiety Disorder 7-item is a validated population-based survey instrument for measuring anxiety symptoms [28] (Cronbach α=.81 in this study sample). Scores of 5, 10, and 15 on the Generalized Anxiety Disorder 7-item correspond to mild, moderate, and severe anxiety, respectively. The Patient-Reported Outcome Measurement Information System (PROMIS) Anger 5a short form [29] assesses self-reported angry mood (eg, irritability; Cronbach α=.82). The PROMIS–Social Isolation 8a [30] contains 8 items that evaluate feelings of being avoided, excluded, detached, or disconnected (Cronbach α=.89). For both PROMIS measures, raw scores were converted into *t* scores, which had a mean of 50 and an SD of 10, using the conversion tables provided [31].

Physical health outcomes, including insomnia and pain, were assessed using 2 measures. Designed as a brief screening tool for insomnia, the Insomnia Severity Index has 7 questions asking about the nature and symptoms of sleep problems [32] (Cronbach α=.78). Scores of 8, 15, and 22 on the Insomnia Severity Index represent thresholds for subthreshold, moderate clinical, and severe clinical insomnia, respectively. The Pain, Enjoyment, General Activity scale includes 3 items measuring the severity of pain and its interference with enjoyment of life and general activity (Cronbach α=.93) [33].

In addition, the EQ-5D-5L is a self-report survey of global health and health-related quality of life, containing questions across 5 domains: mobility, self-care, usual activities, pain or discomfort, and anxiety or depression [34] (Cronbach α=.76). Responses to the EQ-5D-5L were converted into utility values using an algorithm for the US population [35], with a higher score corresponding to better health-related quality of life.

#### Potential Mediators

Potential mediators include the CBT-related mechanisms of change. A total of 14 items from the Cognitive Behavioral Therapy Skills Questionnaire [36] were used for a general assessment of CBT skill acquisition and the use of the 2 core CBT skills: cognitive restructuring and behavioral activation (Cronbach α=.83). The 9-item Behavioral Activation for Depression Scale–Short Form, which includes subscales for activation and avoidance, is often used to track changes in the behaviors hypothesized to underlie depression and specifically targeted for change by behavioral activation [37] (Cronbach α=.80). In addition, the 16-item Basic Needs Satisfaction in General Scale [38] was designed to assess satisfaction with 3 basic psychological needs: autonomy, competence, and relatedness (Cronbach α=.77). Autonomy pertains to an individual’s desire to perceive their actions and subsequent consequences as self-determined, rather than being subject to external influences or control [39]. Competence refers to the inherent desire to experience a sense of efficacy and proficiency in task execution across a range of complexities [40]. Relatedness refers to the inherent human desire for social connection, support, and caring from others [40]. According to the self-determination theory, all 3 needs must be fulfilled to achieve psychological well-being [39].

### Statistical Analysis

Descriptive statistics were computed for the demographic characteristics, psychosocial outcome measures, program usability, acceptability, and engagement measures. To compare baseline characteristics between the groups, chi-squared tests were used for categorical variables, whereas 2-sample 2-tailed *t* tests were used for continuous variables. Paired 2-tailed *t* tests were used to assess within-group differences from baseline to posttest. In addition, 2-sample 2-tailed *t* tests were used to evaluate between-group differences in outcomes at the posttest stage. Linear mixed modeling was further applied to analyze within-group changes over time and compare changes in depressive symptoms between the treatment and control groups.

Causal mediation analysis [41,42] was conducted to explore the mechanisms driving the intervention effects, specifically focusing on the mediators described in the *Potential Mediators* section. Using the *mediate* command in Stata, individual models were estimated separately for each mediator [43]. Potential confounders that might influence both the mediator and the outcome were adjusted in the model, including age, gender, education, income, living arrangement, and count of chronic physical conditions. In addition, to obtain more robust estimates of indirect effects, the baseline PHQ-9 score was adjusted in the outcome equation, and the baseline score of each corresponding mediator was included in the mediator equation.

For analyses related to depressive symptoms, an intention-to-treat design was used by imputing missing values using the last observation carried forward method. Owing to the small sample size, the low incidence of missing values, and the exploratory nature of the secondary aims, analyses related to secondary outcomes and potential mediators were conducted using complete data, without imputation of missing values. Unless otherwise specified, 2-sided tests (α=.05) were used to determine statistical significance. All analyses were performed using Stata SE (version 18; StataCorp LLC).

## Results

### Participants

In total, 70 participants were enrolled and assigned to either the treatment or waitlist attention control group, as depicted in Figure 1. The baseline characteristics of the participants are presented in Table 1. Bivariate analyses revealed no significant differences between the treatment and control groups at baseline (all *P* values were >.05 for 2-tailed *t* tests). The participants mainly consisted of women (57/70, 81%) and were primarily non-Hispanic White (56/70, 80%). More than half held at least a college degree (40/70, 57%) and were unmarried (37/70, 53%). In addition, over half had an annual household income <US $50,000 (40/70, 58%). Most participants (60/70, 86%) owned a laptop or PC. The majority had been diagnosed with depression by a health care professional (44/70, 63%), but only around a third (25/70, 36%) reported taking antidepressants. Notably, nearly all participants (68/70, 97%) indicated some lower body mobility difficulties, with an average of approximately 4 items (mean 4.46, SD 2.53) endorsed on the mobility limitation index.

**Table 1:** Baseline characteristics of the study sample (N=70).

			Control group		Treatment group		Control vs treatment							
							*t* test (*df*)			Chi-square (*df*)			*P* value	
Age (years), mean (SD; range)			67.91 (5.83; 60-85)		69.80 (5.36; 61-86)		–1.41 (68)			N/A^a^			.16	
**Gender, n (%)**								N/A			0.9 (1)			.36
	Female	30 (85)		27 (77)										
	Male	5 (14)		8 (22)										
**Race or ethnicity, n (%)**								N/A			5.4 (4)			.25
	African American or Black	4 (11)		5 (14)										
	American Indian	1 (3)		0 (0)										
	Asian or Pacific Islander	0 (0)		2 (6)										
	White, non-Hispanic	30 (86)		26 (74)										
	Mixed race or other	0 (0)		2 (5)										
**Education, n (%)**								N/A			2.9 (3)			.41
	High school or GED^b^	4 (11)		6 (17)										
	Associate’s or some college	8 (23)		12 (34)										
	4-year college degree	13 (37)		12 (34)										
	Master’s degree or above	10 (29)		5 (14)										
**Household income (US $), n (%)**								N/A			2.5 (3)			.47
	<20,000	2 (6)		5 (15)										
	20,000-49,999	17 (49)		16 (47)										
	50,000-74,999	9 (26)		5 (15)										
	≥75,000	7 (20)		8 (23)										
**Living status, n (%)**								N/A			0.1 (1)			.81
	Lives with others	21 (60)		20 (57)										
	Lives alone	14 (40)		15 (43)										
**Marital status, n (%)**								N/A			4.7 (4)			.30
	Married	18 (51)		13 (37)										
	Living with a partner	1 (3)		1 (3)										
	Divorced or separated	6 (17)		14 (40)										
	Widowed	8 (23)		5 (14)										
	Never married	2 (6)		2 (6)										
Count of chronic conditions^c^, mean (SD; range)			2.31 (0.99; 0-5)		2.25 (1.27; 0-5)		0.2 (68)			N/A			.83	
Mobility limitation index^d^, mean (SD; range)			4.20 (2.29; 1-9)		4.71 (2.76; 0-10)		–0.9 (68)			N/A			.40	
**Device ownership, n (%)**								N/A			0.7 (2)			.69
	No device ownership	2 (6)		2 (6)										
	Has tablet or iPad	4 (11)		2 (6)										
	Has laptop or PC	29 (83)		31 (89)										
**Ever received a depression diagnosis^e^, n (%)**								N/A			0 (1)			.99
	No	13 (37)		13 (37)										
	Yes	22 (63)		22 (63)										
**Taking an antidepressant, n (%)**								N/A			0.6 (1)			.45
	No	21 (60)		24 (69)										
	Yes	14 (40)		11 (31)										
PHQ-9^f^ score, mean (SD; range)			11.29 (2.65; 8-18)		11.49 (2.63; 8-19)		–0.3 (68)			N/A			.75	

^a^N/A: not applicable.

^b^GED: General Educational Development Test.

^c^The chronic disease count was the sum of self-reported conditions including hypertension, diabetes, chronic lung disease, chronic kidney disease, heart disease, stroke, arthritis, and cancer.

^d^The mobility limitation index asks if a participant has difficulty with 11 tasks: running or jogging for a mile, walking several blocks, walking one block, sitting for 2 hours, standing for 30 minutes, getting up from a chair after sitting for a long period, climbing several flights of stairs, climbing 1 flight of stairs, walking across a room, using equipment or devices when crossing a room, and getting in or out of bed. “Yeses” were summed to create a total score, ranging from 0 to 11.

^e^Participants were asked, “has a doctor, therapist, psychologist, or other health care provider ever told you that you have depression?”

^f^PHQ-9: Patient Health Questionnaire-9.

### Primary Clinical Outcome

Table 2 shows the results of the linear mixed effects regression analysis of depressive symptoms. The main effect of time was significant (b=–0.49, 95% CI –0.73 to –0.26; *P*<.001), showing a decrease of nearly 0.5 points on the PHQ-9 for every biweekly assessment time point in the control group. The group-by-time interaction was also significant (b=–0.68, 95% CI –1.00 to –0.35; *P*<.001), which indicates that the treatment group experienced a faster decline in their PHQ-9 score by 0.68 points per assessment time point compared with the control group. Considering both the main and interaction effects, the PHQ-9 score in the treatment group was projected to decrease by 1.17 points every 2 weeks.

**Table 2:** Linear mixed modeling predicting PHQ-9^a^ scores^b^.

		Estimate (95% CI; SE)	*t* test	*P* value
Intercept		10.48 (9.50 to 11.45;0.50)	21.14	<.001
**Group allocation**				
	Control group	Reference		
	Treatment group	1.06 (–0.31 to 2.43; 0.70)	1.52	.13
Time		–0.49 (–0.73 to –0.26; 0.12)	–4.09	<.001
Group×Time		–0.68 (–1.00 to –0.35; 0.17)	–4.09	<.001

^a^PHQ-9: Patient Health Questionnaire-9.

^b^Time codes represent the sequential order of assessments: 0 for the baseline, 1 for the first in-app assessment, and so on, culminating with 6 for the posttest assessment. Each assessment was performed approximately 2 weeks apart.

Effect sizes were calculated using pretest scores to subtract posttest assessment scores so that a positive effect size indicates reduced depressive symptoms as measured by the PHQ-9. The treatment group had a large within-group effect size from baseline to posttest assessment (Cohen *d*=1.22; *P*<.001). By contrast, the waitlist attention control group experienced a medium within-group effect (Cohen *d*=0.57; *P=*.002). Taking everything into account, Empower@Home had a medium between-group effect size compared with waitlist attention control at the posttest (Cohen *d*=0.72; *P=*.004). Descriptive statistics were used to describe clinically significant improvements in the treatment group. Most participants in Empower@Home (21/35, 60%) experienced a clinically meaningful change in depressive symptoms, defined as a reduction of ≥5 points on the PHQ-9. Of those who initially scored ≥10 on the PHQ-9 before treatment, two-thirds (19/ 25, 76%) exhibited a partial response by scoring <10 at posttreatment. Furthermore, almost half of the Empower@Home participants (16/35, 46%) achieved a score <5 on the PHQ-9 at posttest assessment, suggesting remission.

### Coaching

In total, 7 coaches provided 286 coaching calls to 35 participants, with an average of 8.2 (SD 2.1) calls per participant. Calls varied in length from 11 to 92 minutes, with an average of 49 (SD 16.9) minutes each. On the basis of the coaching notes, most participants (168/286, 58.7%) completed web-delivered lessons on their own before their coaching session. The remainder either partially completed or went through an entire web-delivered lesson along with their coach during the session. Notably, calls were shorter for those who completed sessions independently, averaging 41 (SD 16.6) minutes, compared with an average of 59 (SD 10.9) minutes when a coach guided them through the web-delivered sessions.

On the basis of the coach self-reports, the primary form of assistance provided was simple feedback, such as words of encouragement, which was given in 88.5% (253/286) of the coaching calls. This was followed by facilitating the understanding of the lesson content (147/286, 51.4%), assisting with the application of program tools (143/286, 50%), reviewing or aiding with home practices (120/286, 41.6%), and offering technical assistance (30/286, 10.5%).

### Usability, Acceptability, and Engagement Outcomes

Usability, acceptability, and engagement outcomes were only pertinent to participants in the treatment group. Most participants completed all 9 program sessions (31/35, 89%), averaging 8.3 (SD 2.1) sessions.

Regarding usability, the average SUS score was 82 (SD 12.7), exceeding the benchmark score of 68, indicating that participants perceived the program as usable. A vast majority (30/35, 88%) had an SUS score of ≥68, and all participants (35/35, 100%) agreed or strongly agreed that the program was easy to use.

For acceptability, the average TEI score was 45.9 (SD 6.6), surpassing the benchmark score of 32, indicating favorable attitudes toward the treatment. Most participants (30/35, 88%) stated that they would recommend this program to others who experience depressed moods. However, about a quarter of the participants (9/35, 26%) mentioned that they “experienced discomfort during this program.” Further analysis of their feedback from open-ended follow-up questions revealed that most of these responses stemmed from the program evoking distressing emotions or recalling memories from their past. Interestingly, 2 participants clarified that they perceived this discomfort as positive (“good discomfort”), describing it as a constructive force encouraging them to change their behaviors. Furthermore, 3 participants either agreed or strongly agreed that the program had undesirable side effects; analyzing their open-ended feedback mirrored sentiments from the discomfort-related feedback, indicating that the program helped them confront their issues more directly.

### Secondary Clinical Outcomes

Table 3 displays the within- and between-group effect sizes for the secondary outcomes.

**Table 3:** Within-group and between-group differences for secondary outcomes and mediators^a^.

Measure and condition		Baseline, mean (SD)	Posttest assessment, mean (SD)	Within-group difference				Between-group difference at posttest assessment^b^		
				Cohen *d*	*t* test (*df*)	*P* value	Cohen *d* (95% CI)		*t* test (*df*)	*P* value
**GAD-7^c^**										
	Treatment	7.85 (3.73)	5.06 (4.71)	0.60	3.48 (33)	.001	0.04 (–0.45 to 0.54)		0.17 (62)	.87
	Control	6.17 (5.25)	5.23 (3.38)	0.27	1.48 (29)	.15	N/A^d^		N/A	N/A
**PROMIS-anger^e^**										
	Treatment	55.20 (6.52)	50.06 (9.08)	0.65	3.79 (33)	<.001	0.27 (–0.22 to 0.77)		1.48 (62)	.16
	Control	52.67 (7.06)	52.32 (7.14)	0.06	0.34 (29)	.74	N/A		N/A	N/A
**PROMIS-social isolation**										
	Treatment	54.81 (8.62)	52.33 (7.98)	0.41	2.37 (33)	.02	0.31 (–0.18 to 0.80)		1.24 (62)	.22
	Control	53.75 (6.85)	54.48 (5.52)	–0.14	–0.75 (29)	.46	N/A		N/A	N/A
**Insomnia Severity Index**										
	Treatment	13.68 (5.95)	10.74 (6.81)	–0.49	2.88 (33)	.007	0.21 (–0.28 to 0.70)		0.83 (62)	.41
	Control	12.33 (4.58)	12.00 (4.68)	0.09	0.50 (29)	.62	N/A		N/A	N/A
**PEG^f^**										
	Treatment	4.44 (2.79)	3.54 (2.50)	0.39	2.30 (33)	.03	0.35 (–0.14 to 0.84)		1.40 (62)	.17
	Control	4.36 (2.37)	4.38 (2.33)	–0.02	–0.10 (29)	.92	N/A		N/A	N/A
**EQ-5D-5L**										
	Treatment	0.72 (0.21)	0.74 (0.20)	0.12	0.68 (33)	.50	0.33 (–0.15 to 0.82)		1.31 (62)	.20
	Control	0.72 (0.25)	0.66 (0.27)	–0.26	–1.14 (29)	.17	N/A		N/A	N/A
**CBTSQ^g^**										
	Treatment	40.85 (8.73)	50.26 (8.11)	1.11	6.45 (33)	<.001	0.67 (0.17 to 1.17)		2.69 (62)	.009
	Control	44.97 (8.08)	44.90 (7.81)	–0.01	–0.05 (29)	.96	N/A		N/A	N/A
**BADS-SF^h^**										
	Treatment	26.53 (12.22)	34.21 (9.98)	0.64	3.73 (33)	<.001	0.43 (–0.07 to 0.92)		1.70 (62*)*	.09
	Control	27.50 (10.31)	29.77 (10.93)	0.19	1.05 (29)	.30	N/A		N/A	N/A
**BNSG-S^i^**										
	Treatment	62.18 (9.44)	66.91 (8.11)	0.70	4.11 (33)	<.001	0.52 (0.02 to 1.02)		2.08 (62)	.04
	Control	61.0 (8.75)	62.3 (9.64)	0.22	1.23 (29)	.23	N/A		N/A	N/A

^a^There were 34 participants in the treatment group and 30 in the control group. Within-group differences were evaluated using paired 2-tailed *t* tests for each measure or group separately. Between-group differences were evaluated using 2-sample 2-tailed *t* tests for each measure separately. Analyses were conducted using complete data without imputation. Effect sizes were calculated such that a positive effect size indicated changes in the desired direction (ie, increased quality of life, cognitive behavioral therapy skill acquisition, behavioral activation, and basic needs satisfaction and decreased anxiety, pain, insomnia severity, anger, and social isolation).

^b^Between-group difference at posttest assessment is calculated by comparing the treatment and control condition, cells with a dash correspond with nonapplicable values.

^c^GAD-7: Generalized Anxiety Disorder scale, 7-item.

^d^N/A: not applicable.

^e^PROMIS: Patient-Reported Outcome Measurement Information System.

^f^PEG: Pain, Enjoyment, General Activity.

^g^CBTSQ: Cognitive Behavioral Therapy Skills Questionnaire.

^h^BADS-SF: Behavioral Activation for Depression Scale–Short Form.

^i^BNSG-S: Basic Needs Satisfaction in General Scale.

In the treatment group, significant reductions were noted from baseline to posttest in multiple domains: anxiety (Cohen *d=*0.60; t_33_=3.48; *P=*.001), anger (Cohen *d=*0.65; t_33_=3.79; *P*<.001), social isolation (Cohen *d*=0.41; t_33_=2.37; *P=*.02), insomnia severity (Cohen *d*=0.49; t_33_=2.88; *P=*.007), and pain intensity and interference (Cohen *d*=0.39; t_33_=2.30; *P=*.03). By contrast, the control group showed only minor absolute within-group changes, and none of these changes reached statistical significance. Although there were no statistically significant between-group differences in these outcomes owing to the small sample size, the findings were consistent with our expectations that the treatment group would exhibit greater improvements in the secondary outcome measures from baseline to posttest.

The within-group change in health-related quality of life, as measured by EQ-5D-5L, was not statistically significant for either the treatment or the control group. Notably, the treatment group experienced a slight increase in the quality of life, which aligns with our expectations, whereas a decrease was observed in the control group. Although the differences were not statistically significant, the between-group effect was more pronounced than the within-group effect owing to the divergent trends observed in each group.

### Mediation Analysis

As shown in Table 3, within the treatment group, significant increases were observed at posttest assessment from baseline in all 3 mediators: CBT skills acquisition (Cohen *d=*1.11; t_33_=6.45; *P<*.001), behavioral activation (Cohen *d=*0.64; t_33_=3.73; *P*<.001), and satisfaction with basic psychological needs (Cohen *d*=0.70; t_33_=4.11; *P*<.001). By contrast, the control group showed only minor absolute changes within group, and none of these changes reached statistical significance.

At posttest assessment, significant between-group differences were observed in CBT skills acquisition (Cohen *d*=0.67; t_33_=2.69; *P=*.009) and basic needs satisfaction (Cohen *d*=0.52; t_33_=2.08; *P=*.04). Moreover, the between-group difference in behavioral activation approached the critical value (Cohen *d*=0.43; t_33_=1.70; *P=*.09), reaching significance in a 1-tailed test.

As illustrated in Table 4, the indirect effects associated with all 3 mediators were statistically significant. This suggests that the effect of the intervention on depression was partially mediated by an increase in CBT skills acquisition (b=–2.29, 95% CI –3.58 to –1.01; *P<*.001), behavioral activation (b=–1.27, 95% CI –2.39 to –0.15; *P=*.03), and satisfaction with basic psychological needs (b=–1.38, 95% CI –2.45 to –0.31; *P=*.01). The largest proportion of the mediation effect was seen in CBT skills acquisition (62.9%), followed by satisfaction with basic psychological needs (46.8%), and behavioral activation (34.5%). This proportion was calculated as the ratio of the natural indirect effect to the total effect.

**Table 4:** Indirect and direct effects from causal mediation analysis on depressive symptoms at posttreatment (n=64)^a^.

Mediator	Nature indirect effect		Nature direct effect			Proportion mediated	
	b (95% CI)	*P* value	b (95% CI)	*P* value	Percentage, %		*P* value
CBTSQ^b^	–2.29 (–3.58 to –1.01)	<.001	–1.35 (–3.38 to 0.68)	.19	62.9		.005
BADS-SF^c^	–1.27 (–2.39 to –.15)	.03	–2.41 (–4.09 to –0.72)	.005	34.5		.02
BNSG-S^d^	–1.38 (–2.45 to –.31)	.01	–1.57 (–3.36 to 0.23)	.09	46.8		.02

^a^The outcome was depressive symptoms, as measured by the Patient Health Questionnaire-9, with a higher score indicating greater symptoms. The control group was used as the reference in the treatment model. Each mediator was evaluated individually in separate models. Each model included potential confounders, such as age, gender, education, income, living arrangement, and count of chronic physical conditions, in both the mediator and outcome models. Baseline depression was included in the outcome equation, and the baseline score of each mediator was included in the mediator equation. In addition, the outcome equation includes the treatment-mediator interaction. Analyses were conducted using complete data without imputation).

^b^CBTSQ: Cognitive Behavioral Therapy Skills Questionnaire.

^c^BADS-SF: Behavioral Activation for Depression Scale–Short Form.

^d^BNSG-S: Basic Needs Satisfaction in General Scale.

## Discussion

### Principal Findings

This RCT assessed the efficacy of layperson-supported Empower@Home, a wCBT program specifically designed to alleviate depression among older adults, compared with a waitlist attention control group that received weekly friendly calls. The retention rate in the study was excellent, with 91% (64/70) of the participants engaging in the posttest interview, surpassing the 80% benchmark. Moreover, the intervention engagement rate was high, with 89% (31/35) of individuals in the treatment group completing all 9 program sessions. To put this into perspective, the average completion rate of wCBT programs, which is typically defined as completing 80% of treatment lessons, is only 17% for self-administered interventions and 65% for supported interventions [44,45]. In addition, Empower@Home demonstrated a large within-group effect in the treatment group (Cohen *d*=1.22) and a medium between-group effect accounting for the attention control group at posttest assessment (Cohen *d=*0.72). These findings suggest that the novel intervention is acceptable to older adults and more efficacious in alleviating depression in older adults than weekly friendly calls and symptom monitoring.

Several factors may have contributed to the notably higher engagement rate observed with Empower@Home. Supported wCBT programs tend to exhibit better completion rates compared with self-guided programs, as evidenced by previous studies [44,45]. The presence of coaches likely played a crucial role in this regard by enhancing accountability, fostering interactivity within the program, and establishing a routine for session participation. Another potential factor is the increased global demand for mental health treatments, which has gained momentum during the COVID-19 pandemic [46]. Reports from our community partners who serve older adults in Michigan align with these global observations. Participants may have been motivated to complete the program in their pursuit of improved well-being, especially when faced with barriers in accessing alternative treatment options. Furthermore, although a monetary incentive of up to US $100 was available to the participants, it is improbable that this had a substantial impact on program engagement. This is because our research staff consistently emphasized that payments were not contingent on program completion. In addition, it is worth noting that studies offering higher incentive amounts have sometimes seen lower engagement rates compared with our study [9].

The findings concerning the program’s usability, acceptability, and effects observed in this RCT align with those found in a previous uncontrolled study in which an initial version of the program was examined [14]. In comparison with the uncontrolled study, individuals in this RCT were older by an average of 5 years (68.9 vs 63.7), and this RCT had a larger percentage of individuals with at least 3 physical health conditions (31/70, 44% vs 29/103, 28.2%). However, the educational background and income distribution of the participants were similar across studies. More than half of the participants (45/70, 64%) in the RCT were reached through social media advertisements, a method not used in the uncontrolled study. Furthermore, most of the coaches (n=4) involved in the RCT were new and did not participate in the preceding uncontrolled study. Considering all factors, these consistent findings enhance the likelihood that the study results can be replicated under varied conditions.

The layperson-supported Empower@Home program showed a large within-group effect (Cohen *d*=1.22), aligning with previous wCBT trials with older adults, which had a pooled effect size of 1.27 as reported in a meta-analysis evaluating wCBT programs with varying levels of human support [23]. Meanwhile, the between-group effect was medium (Cohen *d*=0.72), which appears smaller than the average of 1.18 reported in the meta-analysis [23]. Notably, none of the controlled trials in the meta-analysis featured a potent attention control condition such as the one used in this study; instead, they used usual care or waitlist control. In this study, the attention control group also showed a significant reduction in depressive symptoms, as indicated by a medium within-group change (Cohen *d*=0.57). Given that empathy-oriented telephone programs administered by lay callers have been shown to improve depression and mental health among older adults [47], the diminished between-group effect observed here, which results from significant improvements in the control group, is understandable. This observation is consistent with a recent RCT involving homebound older adults with depression, in which a tele-delivered behavioral activation treatment conducted by lay counselors demonstrated a medium effect (Cohen *d*=0.62, 95% CI 0.35-0.89) compared with an attention control that received weekly support telephone calls [48].

The clinical efficacy of the intervention was bolstered by the change mechanisms identified through the causal mediation analysis. In CBT, depressive symptoms are believed to result from maladaptive thoughts and behavioral patterns [49]. Consistent with the theory underlying CBT, we found that reductions in depressive symptoms among participants were partially explained by cognitive restructuring and behavioral activation, as measured by the Cognitive Behavioral Therapy Skills Questionnaire and Behavioral Activation for Depression Scale–Short Form. In addition to these well-known change mechanisms inherent in CBT treatment, we found that the treatment also improved satisfaction with basic psychological needs through an enhanced sense of autonomy, competence, and relatedness, which, in turn, contributed to the reduction of depressive symptoms. This finding resonates with the self-determination theory, which underscores the importance of autonomy, competence, and relatedness in fostering intrinsic motivation [50] and posits that fulfilling these needs is essential for attaining psychological well-being.

This study provides evidence that laypersons lacking specialized mental health skills can effectively offer human support in digital mental health interventions such as wCBT programs. Previous research has shown that supported interventions, in which individuals receive human assistance, tend to enhance engagement and adherence, resulting in more significant positive outcomes compared with unsupported interventions [17,18,51]. However, most existing wCBT programs typically involve therapists with specialized mental health training. Given the scarcity of mental health professionals specialized in working with older adults, particularly in underserved areas, a pragmatic approach may involve training laypersons to assist users of wCBT programs. As the core therapeutic components are preprogrammed, the training requirements for wCBT supporters are significantly reduced. This approach makes layperson-supported wCBT a potentially cost-effective and scalable alternative to therapist-supported interventions. Notably, Titov et al [52] showed that layperson-supported wCBT for depression was as effective as a clinician-supported intervention in an RCT involving adults (mean age 44, SD 12.3 years). Although research specific to older adults remains limited, Tomasino et al [9] showed that a wCBT program supported by peers was well received and associated with a significant reduction in depressive symptoms in a small sample of older adults with depression. In another study focusing on low-income homebound older adults, Choi et al [48] reported that behavioral activation treatment administered via telecommunication and led by lay counselors with bachelor’s degrees was more effective than the control condition of weekly support calls. Although the study by Choi et al [48] also found that the lay counselor-led intervention was not as effective as tele-delivered problem-solving therapy conducted by clinicians in alleviating depression, it yielded comparable results in terms of secondary outcomes. These emerging findings provide support for the feasibility and potential cost-effectiveness of having laypersons as human supporters of wCBT programs.

The findings of this study have important practical implications. wCBT programs, such as Empower@Home, can serve as valuable resources for health care professionals working with older adults, including case managers, care coordinators, and primary care physicians. These tools can be especially beneficial for individuals facing persistent treatment barriers such as transportation issues and financial constraints. Although challenges related to technology access and digital literacy may exist, this study shows that with guidance and support, older adults are generally receptive to using wCBT programs. However, it is important to note that wCBT programs should be considered as complementary tools rather than replacements for mental health professionals. They can supplement the traditional one-on-one therapy provided by mental health professionals by offering additional, easily accessible resources for clients to use outside of therapy sessions. wCBT programs can also serve as intermediate support for clients waiting for traditional treatments or as a means to enhance the continuum of care. By recognizing the role of wCBT in augmenting mental health care, practitioners across various health care disciplines can better address the mental health needs of their clients, especially in underserved or marginalized communities.

### Limitations

This study was limited by its small sample size, rendering it underpowered for the detection of small effect sizes. Although our study was powered to detect a medium effect in the primary clinical outcome of depression based on a linear mixed effects model with up to 7 assessments [3], it was not powered to detect a small effect in the secondary clinical outcomes based on 2-sample 2-tailed *t* tests. We performed an ad hoc power analysis and found that a sample size of 204 is necessary to identify an effect size of 0.35 with a power of 80%, using a 2-sample 2-tailed *t* test, as per G*Power 3.1.9.7 [53]. Moreover, without long-term follow-up, it is unclear whether the treatment effect observed at posttest assessment would be sustained in the long term. In addition, when considering the generalizations of the study findings, it is important to take into account the sources of participant recruitment and their characteristics. Although participants came from across Michigan, including metropolitan and rural areas, their education levels and technology device ownership exceeded the national and state averages among older adults [54,55]. The overrepresentation of college-educated and technology-savvy participants was not unexpected because most participants were recruited from social media advertisements and a research volunteer registry that required internet access.

### Conclusions

wCBT, tailored specifically for older adults and augmented with support from trained laypersons, is efficacious in reducing depressive symptoms compared with friendly telephone calls and symptom monitoring. The intervention works by enhancing several change mechanisms, including facilitating the acquisition of CBT skills, promoting behavioral activation, and fostering self-determination, which manifests as a heightened sense of autonomy, competence, and relatedness. Future well-designed trials are needed to evaluate the effectiveness of the intervention in community and practice settings, leveraging nonclinician staff already present in these real-world settings as wCBT coaches.

## Appendix

Multimedia Appendix 1 CONSORT eHEALTH checklist (V 1.6.1)

## Abbreviations

CBT: cognitive behavioral therapy
PHQ-9: Patient Health Questionnaire-9
PROMIS: Patient-Reported Outcome Measurement Information System
RCT: randomized controlled trial
SUS: System Usability Scale
TEI: Treatment Evaluation Inventory
wCBT: web-delivered cognitive behavioral therapy
